# Regulatory network in lignin biosynthetic pathway through small RNAs in *Acacia mangium*: implications to the pulp and paper industry

**DOI:** 10.1186/1753-6561-5-S7-P120

**Published:** 2011-09-13

**Authors:** Seong Siang Ong, Ratnam Wickneswari

**Affiliations:** 1 School of Environmental and Natural Resource Sciences, Faculty of Science and Technology, Universiti Kebangsaan Malaysia, 43600 Bangi, Selangor, Malaysia

## Background

Lignin, after cellulose, is the second most abundant biopolymer accounting for approximately 15-35% of the dry weight of wood. As an important component of wood, lignin is indispensable for plant structure and defense. However, it is an undesirable component in the pulp and paper industry. Removal of lignin from cellulose is a costly and environmentally hazardous process. Tremendous efforts have been devoted to understand the role of enzymes and genes controlling the amount and composition of lignin to be deposited in the cell wall. However, studies on the impact of downregulation and overexpression of monolignol biosynthesis genes in model species on lignin content, plant fitness and viability have been inconsistent. Recently, non-coding RNAs have been discovered to play an important role in regulating the monolignol biosynthesis pathway genes [1-3]. Non-coding RNAs represent an emerging class of riboregulators, which are processed to shorter miRNAs or siRNAs. The current paradigm indicated that plant system use small RNAs (miRNAs and siRNAs) as guide for post-transcriptional gene silencing and epigenetic regulation. Although miRNAs and siRNAs result from different biogenesis pathways but both interact with target transcripts for direct cleavage or translation repression, effectively shutting down that genes’ functions. However, much less is known about the mechanism of gene regulation governed by these small RNAs in lignin biosynthesis pathway in *A. mangium*.

## Methods

Total RNA was isolated from secondary xylem tissue with contrasting lignin content using mirVana microRNA Isolation Kit (Cat. AM1561, Ambion, Austin, TX, USA) following manufacturer’s protocol. Thin cookies were first ground in a blender and then further ground to fine powder using mortar and pestle. Integrity of the isolated Total RNA was analyzed using Bioanalyser 2100 (Agilent Technology, Palo Alto, USA) and only Total RNA with RIN value above 7 was selected for library construction. Library construction, sequencing and bioinformatics pipeline analysis was done by Gene Pool Sequencing Centre, United Kingdom. Small RNA libraries were generated using DGE small RNA Sample Preparation Kit (Cat. # FC-102-1009; Illumina, San Diego, CA, USA). Illumina sequencing libraries were prepared using the ‘long’ IIIumina protocol according to the manufacturer’s direction and two libraries were sequenced on an IIIumina GA-II following manufacturer’s instructions. After masking of adapter sequences and removal of contaminated reads, the clean reads were filtered and the resulting oligos were totalled. Clustering based on relative lengths from 7 nt to 35 nt were done using in house perl scripts. The targets were extracted by in house python script and annotated using BLAST to GO database. Only highly conserved miRNAs with strong differences in their expression level between high and low lignin secondary xylem were selected for validation using IQ5 real time – PCR technology (BioRad, Hercules, USA) (Table 1).

**Table 1:** Total Counts of the four selected highly conserved miRNA families isolated from secondary xylem of low lignin *A. mangium* 54 (Am54) and high lignin *A. mangium* 48 (Am48).

miRNA family	AM54	Am48	Predicted Target
amg-miR159	3158	1008	MYB Trabscription Factor
amg-miR168	79040	33447	Agrgonuate
amg-miR172	32743	18204	APETALA 2-LIKE transcription factor
amg-miR394	2037	226	F-box

## Results

**Figure 1:**
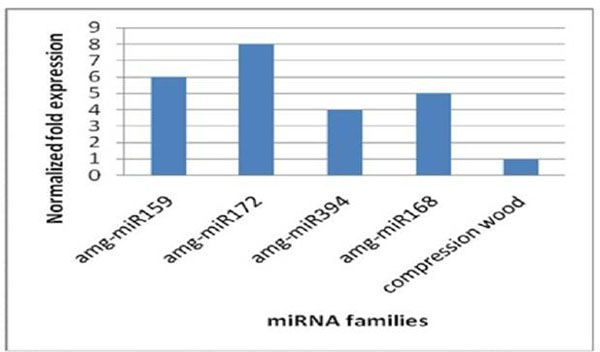
Relative normalized expression of four selected conserved miRNAs in secondary xylem tissue of *A. mangium* Tension Wood (TW) using real time PCR technology. 5.8S rRNA was selected as references genes.

A total of 14,582,383 reads were generated in Am54 and 10,281,313 reads in Am48.We have identified several conserved and novel small RNAs that may serve as an important regulatory role during secondary wall formation. Majority of these small RNAs emerged as critical regulators for normal growth and developmental processes in *A. mangium*. Only a few small RNAs were postulated to play an important role during epigenetic silencing. We found that the expression level of these miRNAs belong to four different families was up regulated in tension wood (Fig. 1). Tension wood is composed almost entirely of cellulose while compression wood is rich in lignin. We will further investigate the effects of over expression of these four highly conserved miRNAs in tension wood on the expression level of monolignol biosynthetic pathway genes.

## Conclusions

From the sequence results, we concluded that* A. mangium* small RNAs consist of a set of 14 highly conserved miRNAs families found in plant miRNA database, 82 novel miRNAs and a large proportion of non-conserved small RNAs with low expression levels. Out of these 14 highly conserved miRNAs families, only four miRNAs families were selected for validation in compression wood and tension wood and their total relative counts between Am54 and Am48 are shown (Table 1). Although these four miRNAs belong to different families, all of them were up regulated in tension wood, a region composed entirely of cellulose. The results obtained can be used to better understand the roles of small RNAs and for the development of a gene constructs for silencing of specific genes involved in monolignol biosynthesis with minimal effect on plant fitness and viability.
